# Supplementary material for: Micronutrients Deficiency, Supplementation and Novel Coronavirus Infections—A Systematic Review and Meta-Analysis
Source: Nutrients. 2021 May 10;13(5):1589. doi: 10.3390/nu13051589 (PMC8151981; doi:10.3390/nu13051589)
Supplement: Supplementary file 1 [file nutrients-13-01589-s001.zip › Supplementary Tables and Figures/Supplementary Tables.pdf]

Table S1. PICOS/PEOS table defining research question in this review

Focus	Prevention	Treatment
Population	Adults >19 years old, not infected with SARS, MERS or COVID-19	Patients >19 years, diagnosed with SARS, MERS or COVID-19 episode
Intervention, Exposure	Supplementation or deficiency of any micronutrient singly, or in combination with other micronutrients	Treatment with any micronutrient singly, or in combination with other i) micronutrients or ii) therapeutic drug or treatment protocol
Comparator	No supplementation or deficiency in micronutrient, as appropriate to intervention/exposure arm	
Outcome	Incidence of SARS, MERS or COVID-19 episode	Severity of COVID-19 episode in terms of: mortality, ICU admission, length of stay, progression to respiratory-related complications and clinical severity
Study Design	Randomised controlled trial, cohort, cross sectional and case-control	

Table S2. Population characteristics in detail

Author, Year [Ref.]	Reported Study Design	Country of Study: Period of data collection	Population source	Description of comorbidities	% men (%)	Mean age (SD)
Baktash, 2020	Prospective cohort	UK: 1 Mar-30 Apr 2020	Emergency admissions aged ⩾65 years admitted to District general hospital	hypertension (n=54), diabetes mellitus (n=34), ischaemic heart disease (n=26), chronic respiratory disease (n=17), heart failure (n=17), stroke (n=12), chronic kidney disease (n=22), atrial fibrillation (n=22), cancer (n=5), dementia (n=7) and endocrinological disease (n=5)	54.3	Cases (deficient): 79.46 (89.52)<br>Cases (non-deficient): 81.16 (7.23)<br>Controls: 83.44 (8.08)
Bellmann-Weiler, 2020	Retrospective cohort	Austria: 25 Feb-20 May 2020	Inpatients with PCR-confirmed SARS-CoV-2 infections at the Innsbruck University Hospital or Hospital St. Vinzenz Zams	CVD (n=152), hypertension (n=124), coronary artery disease (n=35), chronic heart failure (n=9), diabetes mellitus (n=45), CKD (n=15), malignancies (n=21), COPD (n-23) and bronchial asthma (n=9)	60.6	68 (53-80)*
Carpagnano, 2020	Retrospective cohort	Italy: 11 Mar-30 Apr 2020	Consecutive hospitalized adult inpatients with laboratory-confirmed COVID-19 infection, acute respiratory failure and no need of intubation or invasive ventilation in Intensive Care Unit (ICU), admitted to the Respiratory Intermediate Care Unit (RICU) of the Hospital Policlinic of Bari, Italy	hypertension (n=26), CVD (n=16), CKD (n=16), diabetes mellitus (n=11), cerebrovascular disease (n=5), malignancy (n=5), COPD (n=5) and asthma (n=2)	71.4	≥30 ng/mL: 64 (18)<br>30 >Vitamin D ≥20 ng/mL: 64 (13)<br>20 > Vitamin D ≥10 ng/mL: 60 (6.9)<br><10 ng/mL: 74 (11)
D'Avolio, 2020	Retrospective cohort	Switzerland: 1 Mar-14 Apr 2019, 1 Mar-14 Apr 2020	patients who underwent a nasopharyngeal swab PCR analysis for SARS-CoV-2 at Canton of Tessin, Switzerland in 2020	NR	54.2	73 (63-81)*

			1377 controls, without a SARS-CoV-2 PCR test, with at least one measurement for 25(OH)D in the corresponding period (1 March to 14 April) of 2019	NR	45.3	63 (46-76)*
Dahan, 2020	Cross-sectional	Israel: 21 Feb-30 Mar 2020	hospitalised patients with laboratory-confirmed COVID-19 from two hospitals	Prior diagnosis of at least one condition: COPD, diabetes mellitus, dyslipidemia, hypertension, inflammatory bowel disease, ischemic heart disease, multiple myeloma, lymphoma, rheumatoid arthritis, scleroderma	59	52.46 (2.76)
Hastie, 2020	Retrospective cohort	UK: 2006-2010 (exposure measurement), 5 Mar-25 Apr 2020 (outcome measurement)	UK Biobank participants aged 37–73 years between 2006 and 2010, with baseline serum 25(OH)D concentration and complete data on covariates with COVID-19 mortality linked to the Death Register	A portion of the participants were with chronic diseases (unspecified); NR;	NR	37–73 (between 2006-2010)[#]
Im, 2020	Case-control	South Korea: Feb-Jun 2020	<u>Cases:</u><br>Adults (>15 years old) with laboratory-confirmed COVID-19 infection admitted to Inha University Hospital<br><u>Controls:</u><br>Patients who were tested for 25-hydroxyvitamin D3 in February–June, the same season in which COVID-19 patients presented to the hospital, assigned 3:1 to each COVID-19 patient after age- and sex-matching	Cases and Controls: NR	Cases: 42<br>Controls: NR	Cases: 57.5 (34.5-68)<br>Controls: NR
Jothimani, 2020	Prospective case-control	India: 17 May-27 May 2020	<u>Cases:</u>	Cases: NR<br>Controls: no underlying comorbidities	Cases: 61.5	Cases: 34 (18-77)*

			Laboratory-confirmed COVID-19 patients referred to the Dr. Rela Institute and Medical Centre<br>Controls:<br>Hospital staff members from the outpatient department		Controls: 67.7	Controls: 32 (18-60)*
Karahan, 2020	Retrospective cohort	Turkey: 1 Apr-20 May 2020	Adult COVID-19 patients in Health Sciences University, Bagcilar Training and Research Hospital, Istanbul, Turkey.	coronary artery disease (n=32), hypertension (n=85), dyslipidemia (n=39), diabetes mellitus (n=61), COPD (n=15), malignancy (n=23), CKD (n=29), congestive heart failure (n=35), acute kidney injury (n=16) and chronic atrial fibrillation (n=15)	54.4	63.5 (15.3)
Liu, 2020	Retrospective cohort	China: 9 Feb-15 Feb 2020 (follow-up till 25 Feb 2020)	Severe COVID-19 patients admitted in Tongji Hospital of Tongji Medical College, Huazhong University of Science and Technology, defined according to the diagnostic and treatment guideline criteria issued by Chinese National Health Committee (Version 3-5)	Hypertension (n=40), diabetes mellitus (n=20), coronary heart disease (n=11), respiratory system diseases (n=11), cerebrovascular diseases (n=10)	49	68 (61-76)*
Macaya, 2020	Retrospective cohort	Spain: 5 Mar-31 Mar 2020	Laboratory-confirmed COVID-19 cases attending the emergency department at a tertiary hospital in Madrid	Hypertension (n=50), diabetes mellitus (n=32), cardiac disease (n=19), advanced chronic kidney disease (n=26), chronic respiratory disease (n=13) and obesity (n=23)	43.8	non-severe patients: 63 (50-72)*<br>severe patients: 75 (66-84)*
Maghbooli, 2020	Cross-sectional	Iran: Till 1 May 2020 (start date unspecified)	CT-confirmed or laboratory-confirmed patients (>= 18 years old) at Sina Hospital, Tehran	Acute respiratory tract infection symptoms and a history of chronic disorders (n=155)	61.3	58.72 (15.22)
Mardani, 2020	Cross-sectional	Iran: Mar 2020	Patients (18-78 years old) during March 2020 with respiratory difficulties including cough and shortness of	chronic lung diseases, hematological diseases, liver disease, having undergone radiotherapy, and chemotherapy were excluded from the study.	52.8	42 (NR)

			breath and/or with CT-scan showing ground glass opacity in lungs in clinic			
Meltzer, 2020	Retrospective cohort	United States: 3 Mar-10 Apr 2020	patients who tested for COVID-19 at the University of Chicago Medicine, with Vitamin D level measurements taken within 1 year but not within the 14 days from COVID-19 testing	Hypertension (n=261), diabetes (n=137), chronic pulmonary disease (n=117), pulmonary circulation disorder (n=20), depression (n=119), chronic kidney disease (n=116), liver disease (n=56), comorbidities with immunosuppression (n=105)	25	49.2 (18.4)
Merzon, 2020	Retrospective cohort	Israel: 1 Feb-30 Apr 2020	Patients from Leumit Health Services who tested for COVID-19 and had at least one previous blood test for plasma 25(OH) level	Depression/anxiety (n=890), schizophrenia (n=156), dementia (n=454), diabetes (n=1732), hypertension (n=2136), cardiovascular disease (n=1250), chronic lung disorder (n=1001), obesity (n=1925)	41.4	COVID-19 positive: 35.58 (0.56)<br>COVID-19 negative: 47.35 (0.24)
Moghaddam, 2020	Cross-sectional	Germany: NR	RT-PCR confirmed COVID-19 patients from the non-profit Public Hospital	Hypertension (n=22), diabetes (n=6), chronic obstructive pulmonary disease (n=1), cardiovascular disease (n=17), cerebrovascular disease (n=6) and obesity (n=7)	42.4	77 (38-94)*
Panagiotou, 2020	Cross-sectional	UK: NR	Patients with laboratory, clinical or radiological diagnosis of COVID-19, admitted in Tyne Hospitals in Newcastle	114 patients with ≥1 comorbidity including hypertension (n=56), diabetes (n=38), obesity (n=14), malignancy (n=15), respiratory (n=42), CVD (n=20), kidney and liver disease (n=19) and others (n=14)	54.5	severe: 61.1 (11.8)<br>mild: 76.4 (14.9)
Pizzini, 2020	Prospective cohort	Austria: From 29 Apr 2020 (end date unspecified)	Inpatients and outpatients ⩾18 years with a confirmed infection with SARS-CoV-2 - based on typical clinical presentation and a positive SARS-CoV-2 real-time PCR test in Innsbruck, with two additional participating centers in Zams and Münster, all care centres in Tyrol	109. 88 patients with ≥1 comorbidity including CVD (n=44), hypertension (n=32), pulmonary disease (n=21), endocrine disease (n=49), hypercholesterolemia (n=24), type 2 diabetes (n=20), CKD (n=7), chronic liver disease (n=6), malignancy (n=16) and immunodeficiency (7)	60	58 (14)

Radujkovic, 2020	Prospective cohort	Germany: 18 Mar-18 Jun 2020	Consecutive symptomatic SARS-CoV-2-positive patients admitted to the Medical University Hospital Heidelberg with serum samples available for VitD status analysis	77 patients with comorbidity including CVD (n=58), diabetes (n=19), CKD (n=8), chronic lung disease (n=15) and active/history of malignancy (n=17)	51	60 (49-70)*
Raisi-Estabragh, 2020	Prospective cohort	UK: 2006-2010 (exposure assessment), 16 Mar-18 May 2020	Individuals aged 40 -69 in the National Health Service registers, living within 10 miles of one of 22 UKB (UK Biobank) assessment centres in 2006-2010 and with COVID-19 results available between 16 March - 18 May 2020	Diabetes (n=666), hypertension (n=2,081) and high cholesterol (n=1,471)	48.8	COVID-19 positive: 68.11 (9.23) COVID-19 negative: 68.91 (8,72)
Smith, 2020	Retrospective multi-centre cohort	United States: 1 May-30 Mar 2020	Patients from 8 hospital systems in NYC with known history of gynecologic cancer who were hospitalized for COVID-19 infection and had available inflammatory markers data on admission	36 patients were with <3 comorbidities, 50 patients were with ≥3 comorbidities	0	68.5 (59-74.8)
Sonnweber, 2020	Prospective multi-centre cohort	Austria: NR	Patients aged ≥18 years with previous mild to critical COVID-19 episode	88 patients with ≥1 comorbidity including CVD (n=44), hypertension (n=32), pulmonary disease (n=21), endocrine disease (n=49), hypercholesterolemia (n=24), diabetes (n=20), CKD (7), chronic liver disease (n=6), malignancy (n=16) and immunodeficiency (n=9).	+0	58 (14)
Sun, 2020	Retrospective cohort	China: NR	RT-PCR confirmed non-imported COVID-19 patients in Beijing	Hypertension (n=12), diabetes (n=5), cerebral infarction (n=2), cardiac arrthymia (n=2), prostate cancer (n=1), bronchial asthma (n=2), pulmonary tuberculosis (n=1), thyroid disease (n=3), liver disease (n=2) and chicken pox (n=1)	58.7	Median: 47 (Range: 3-85)

Wu, 2020	Retrospective cohort	China: 25 Dec 2019-26 Jan 2020 (follow-up till 13 Feb 2020)	Patients aged 21 to 83 years with confirmed COVID-19 pneumonia hospitalized at Jinyintan Hospital in Wuhan	Hypertension (n=39), diabetes (n=22), CVD (n=8), liver disease (n=7), nervous system disease (n=7), chronic lung disease (n=5), CKD (n=2), endocrine system disease (n=2) and tumor (n=1)	63.7	51 (43-60)*
Yasui, 2020	Retrospective cohort	Japan: 24 Mar-24 May 2020	Patients with COVID-19 who were admitted to the Sakai City Medical Centre	Diabetes (n=13), CKD (n=6), liver disease (n=3), hyperlipidemia (n=7), CVD (n=2), hypertension (n=20) and respiratory disease (n=8)	54.8	NR, but 17 (27.4%) are aged ≥65 years
Ye, 2020	Case-control	China: 16 Feb-16 Mar 2020	Cases: Patients with COVID-19 disease treated at the Yongwu Hospital of The People's Hospital of Guangxi Zhuang Autonomous Region, Nanning	diabetes (n=5), hypertension (n=6), liver injury (n=1), COPD (n=1) and renal failure (n=16)	37	43 (32-59)*
			Controls: Patients from The Physical Examination Centre of our Institution with no medical disorder, frequency matched by sex and age as the case	Nil	42	42 (31-52)*
Zhao, 2020	Retrospective cohort	China: 1 Feb-29 Feb 2020	hospitalized patients with confirmed COVID-19	Hypertension (n=17), diabetes (n=6), chronic pulmonary disease (n=2), coronary heart disease (n=6), cerebrovascular artherosclerosis (n=2), malignancy (n=1) and chronic liver disease (n=1)	60	55 (44-66)*
Zhou, 2020	Retrospective cohort	China: 29 Dec 2019-31 Jan 2020	inpatients (⩾18 years old) in Jinyintan Hospital and Wuhan Pulmonary Hospital with laboratory confirmed COVID-19 and definite clinical outcome	91 patients with ≥1 comorbidity including hypertension (n=58), diabetes (n=36), coronary heart disease (n=15), chronic obstructive lung disease (n=6), carcinoma (n=2), CKD (n=2) and other (n=22)	62.3	Survivor: 52 (45-58)* Non-survivor: 69 (63-76)*
Annweiler, 2020	Retrospective cohort	France: Mar-Apr 2020	Residents with clinically obvious or RT-PCR-confirmed COVID-19 infection at nursing home	All patients with physical disabilities, major neurocognitive and psychiatric disorders	22.7	87.7 (9.0)

Capone, 2020	Retrospective cohort	United States: till 20 Apr 2020 (start date unspecified)	Patients with confirmed COVID-19 infection at the Brooklyn Hospital Center	Diabetes (n=50), hypertension (n=61), 12 with asthma (n=12), vascular events (prior cerebrovascular accident, coronary artery disease, myocardial infarction, pulmonary embolism, deep vein thrombosis, peripheral arterial disease, HbSC, sickle cell disease; n=30), chronic kidney disease (n=11), HIV (n=5) and gastroesophageal reflux disease (n=10)	53.9	63.22 (53.3-74.3)*
Castillo, 2020	Open-label, double-blind randomised controlled trial	Spain: NR	Patients hospitalized in Reina Sofia University Hospital, with radiographic pattern of viral pneumonia and a positive SARS-CoV-2 PCR with CURB65 severity scale (recommending hospital admission in case of total score > 1)	Hypertension (26), type 2 diabetes (8), lung disease (6), CVD (3) and immunosuppressed and transplanted (7)	58.2	Overall: 53 (10)<br><br>Supplemented group: 53.14 (10.77)<br>Non-supplemented group: 52.77 (9.35)
Fasano, 2020	Retrospective, single-center case-control	Italy: NR	Parkinson's patients living in Lombardy, with at least one evaluation at the Parkinson institute in 2019	hypertension (n=579), chronic obstructive pulmonary disease (n=30), diabetes (n=119), cancer (n=46) and obesity (n=170)	56.9	Case: 70.5 (10.1)<br>Controls: 73.0 (9.5)
Tan, 2020	Retrospective cohort	Singapore: 15 Jan-15 Apr 2020	Consecutive COVID-19 patients ⩾50 years of age admitted to Singapore General Hospital	diabetes (n=6), hypertension (n=24), hyperlipidemia (n=20), cardiovascular disease (n=7), asthma/chronic obstructive pulmonary disease (n=4) and stroke (n=2)	60.4	Non-supplemented: 64.1 (7.9)<br>Supplemented: 58.4 (7)

NA, Not Applicable; NR, Not Reported; UK, United Kingdom; CKD, chronic kidney disease; CVD, cardiovascular disease; COPD, chronic obstructive pulmonary disease; ARDS, acute respiratory distress syndrome

*median (IQR); [#]range

[a] All patients received hydroxychloroquine, antibiotics, and multivitamins, including vitamin C 500 mg twice a day and zinc 150 mg once a day (after the test)

Table S3. Cohort or cross-sectional studies: Detailed breakdown on methodological quality assessment

First Author	Q1	Q2	Q3	Q4	Q5	Q6	Q7	Q8	Q9	Q10	Q11	Q12	Q13	Q14	S	M	D	C	O	Overall
Annweiler, C.	Yes	Yes	Yes	Yes	No	Yes	Yes	No	Yes	No	Yes	CD	Yes	Yes	High	High	Fair	High	Low	Low
Baktash V.	Yes	Yes	Yes	Yes	No	Yes	Yes	Yes	Yes	No	Yes	CD	Yes	No	High	High	Fair	Low	Low	Low
Bellmann-Weiler, R.	Yes	Yes	CD	Yes	No	Yes	Yes	Yes	Yes	No	Yes	CD	Yes	Yes	High	High	Fair	High	Low	Low
Capone, S.	Yes	Yes	Yes	Yes	No	Yes	Yes	No	Yes	CD	Yes	CD	Yes	No	High	High	Fair	Low	Low	Low
Carpagnano, G.E.	Yes	Yes	CD	Yes	No	Yes	Yes	Yes	Yes	No	Yes	CD	Yes	No	High	High	Fair	Low	Low	Low
D'Avolio, A.	Yes	Yes	CD	Yes	No	No	Yes	Yes	Yes	No	Yes	CD	Yes	Yes	High	High	Fair	High	Low	Low
Dahan, S.	Yes	Yes	CD	Yes	No	CD	Yes	Yes	Yes	No	Yes	CD	N/A	No	High	High	Fair	Low	Low	Low
Hastie, C.E.	Yes	Yes	CD	Yes	No	Yes	Yes	Yes	CD	No	Yes	CD	N/A	Yes	High	High	Fair	High	Low	Low
Karahan, S.	Yes	Yes	CD	Yes	No	Yes	Yes	Yes	Yes	No	Yes	CD	Yes	Yes	High	High	Fair	High	Low	Low
Liu, J.	Yes	Yes	CD	Yes	No	Yes	Yes	Yes	Yes	No	Yes	CD	Yes	Yes	High	High	Fair	High	Low	Low
Macaya, F.	Yes	Yes	Yes	Yes	No	Yes	Yes	Yes	Yes	No	Yes	CD	Yes	Yes	High	High	Fair	High	Low	Low
Maghbooli, Z.	Yes	Yes	Yes	Yes	No	Yes	Yes	Yes	Yes	No	Yes	CD	Yes	Yes	High	High	Fair	High	Low	Low
Meltzer, O.	Yes	Yes	Yes	Yes	No	Yes	Yes	Yes	Yes	No	Yes	CD	N/A	Yes	High	High	Fair	High	Low	Low
Merzon, E.	Yes	Yes	Yes	Yes	No	CD	Yes	Yes	Yes	No	Yes	CD	N/A	Yes	High	High	Fair	High	Low	Low
Moghaddam, A.	Yes	Yes	CD	Yes	No	Yes	Yes	Yes	Yes	Yes	Yes	CD	Yes	Yes	High	High	Fair	High	Low	Low
Panagiotou, G.	Yes	Yes	CD	Yes	No	CD	Yes	Yes	Yes	No	Yes	CD	Yes	Yes	High	High	Fair	High	Low	Low
Pizzini, A.	Yes	Yes	CD	Yes	No	No	Yes	Yes	Yes	No	Yes	CD	Yes	No	High	High	Fair	Low	Low	Low
Radujkovic, A.	Yes	Yes	CD	Yes	No	Yes	Yes	Yes	Yes	No	Yes	CD	Yes	Yes	High	High	Fair	High	Low	Low
Raisi-Estabragh, Z.	Yes	Yes	CD	Yes	No	Yes	Yes	Yes	Yes	No	Yes	CD	N/A	Yes	High	High	Fair	High	Low	Low
Smith, M.	Yes	Yes	Yes	Yes	No	Yes	Yes	Yes	Yes	No	Yes	CD	Yes	No	High	High	Fair	Low	Low	Low
Sonnweber, T.	Yes	Yes	Yes	Yes	No	CD	Yes	Yes	Yes	No	Yes	CD	Yes	No	High	High	Fair	Low	Low	Low
Sun, Y.	Yes	No	CD	CD	No	CD	Yes	Yes	Yes	No	Yes	CD	CD	No	Fair	High	Fair	Low	Low	Low
Tan, C, W.	Yes	Yes	Yes	Yes	No	Yes	Yes	No	Yes	N/A	Yes	CD	Yes	Yes	High	High	Fair	High	Low	Low
Wu, C.	Yes	Yes	Yes	Yes	No	Yes	Yes	Yes	Yes	No	Yes	CD	Yes	No	High	High	Fair	Low	Low	Low
Yasui, Y.	Yes	Yes	CD	Yes	No	Yes	Yes	Yes	Yes	Yes	Yes	CD	Yes	Yes	High	High	Fair	High	Low	Low
Zhao, K.	Yes	Yes	CD	Yes	No	CD	Yes	Yes	Yes	Yes	Yes	CD	Yes	No	High	High	Fair	Low	Low	Low
Zhou, F.	Yes	Yes	Yes	Yes	No	Yes	Yes	Yes	Yes	CD	Yes	CD	Yes	Yes	High	High	Fair	High	Low	Low

CD, Cannot determine; N/A, Not applicable; S, selection bias; M, Misclassifiation bias; D, Detection bias; C, Confounding bias; O, Other bias (Inappropriate Sample size, Attrition bias)

Table S4. Case-control studies: Detailed breakdown on methodological quality assessment

First Author	Q1	Q2	Q3	Q4	Q5	Q6	Q7	Q8	Q8	Q10	Q11	Q12	S	M	D	C	O	Overall
Fasano, A.	Yes	Yes	No	Yes	Yes	Yes	N/A	CD	CD	No	No	Yes	High	Low	Low	High	Low	Low
Im, J.	Yes	Yes	No	Yes	Yes	Yes	CD	No	No	Yes	No	No	High	Low	Low	Low	Low	Low
Jothiman, D.	Yes	Yes	No	No	CD	No	CD	CD	No	Yes	Yes	No	Fair	Low	High	Low	Low	Low
Mardani, R.	Yes	Yes	No	Yes	Yes	Yes	CD	No	No	Yes	CD	No	High	Low	Fair	Low	Low	Low
Ye, K.	Yes	Yes	No	CD	CD	No	NA	CD	Yes	Yes	CD	Yes	Fair	High	Fair	High	Low	Low

CD, Cannot determine; N/A, Not applicable; S, selection bias; M, Misclassifiation bias; D, Detection bias; C, Confounding bias; O, Other bias (Inappropriate Sample size, Attrition bias)

Table S5. Randomised Controlled Trial: Detailed breakdown on risk-of-bias assessment for Castillo et al. (2020)

Risk of Bias arising from / Question in Domain	1. From the randomization process	2. Deviations from the intended interventions (effect of assignment to intervention)	3. Missing outcome data	4. Measurement of the outcome	5. Selection of the reported result	Overall
1	Y	PY	Y	N	NI	
2	Y	PY	NA	N	NI	
3	N	NI	NA	PY	NI	
4		NA	NA	PN		
5		NA		NA		
6		Y				
7		NA				
Risk-of-Bias Assessment	Low	Some concerns	Low	Low	Some Concerns	Some Concerns

Y, Yes; PY, Probably Yes; N, No; PN, Probably No; NI, No information; NA. Not Applicable

Table S6. Search strategy used in each database

<table>
<tr><th>Database</th><th>Search Strategy</th></tr>
<tr><td>PubMed</td><td>(((((("deficiency"[MeSH Subheading] OR "deficien*"[Title/Abstract]) OR ("dietary supplements"[MeSH Terms] OR "supplement*"[Title/Abstract])) AND ((((((((((((((("micronutrients"[MeSH Terms] OR "micronutrient*"[Title/Abstract]) OR ("minerals"[MeSH Terms] OR "mineral*"[Title/Abstract])) OR ((((((((((("Zinc Compounds"[MeSH Terms] OR "Zinc"[Title/Abstract]) OR "Selenium"[Title/Abstract]) OR "Selenite"[Title/Abstract]) OR "Selenate"[Title/Abstract]) OR "Selenocysteine"[Title/Abstract]) OR "Selenomethionine"[Title/Abstract]) OR "selenium yeast"[All Fields]) OR "selenium enriched yeast"[All Fields]) OR "magnesium"[Title/Abstract]) OR "Copper"[Title/Abstract]) OR "cupric"[Title/Abstract]) OR "iron"[Title/Abstract])) OR ((("vitamin*"[Title/Abstract] OR "ergocalciferol"[Title/Abstract]) OR "cholecalciferol"[Title/Abstract]) OR "dihydrotachysterol"[Title/Abstract])) OR "beta carotene"[Title/Abstract]) OR "tocopherol"[Title/Abstract]) OR "tocotrienol"[Title/Abstract]) OR "Thiamine"[Title/Abstract]) OR "Riboflavin"[Title/Abstract]) OR "Niacinamide"[Title/Abstract]) OR "Pantothenic acid"[Title/Abstract]) OR "Biotin"[Title/Abstract]) OR "Folic acid"[Title/Abstract]) OR "ascorbic acid"[MeSH Terms]) OR "ascorb*"[Title/Abstract]) OR "cevitamic acid"[Title/Abstract])) OR ("Magnesium deficiency"[MeSH Terms] OR "Avitaminosis"[MeSH Terms])) AND ((((((("middle east respiratory syndrome coronavirus"[MeSH Terms] OR "middle east respiratory syndrome coronavirus"[Title/Abstract]) OR "MERS"[Title/Abstract]) OR "MERS-CoV"[Title/Abstract]) OR "middle east respiratory syndrome related coronavirus"[Title/Abstract]) OR "middle east respiratory syndrome related coronavirus"[Title/Abstract]) OR "middle east respiratory syndrome coronavirus"[Title/Abstract]) OR (((((("sars virus"[MeSH Terms] OR "severe acute respiratory syndrome virus"[Title/Abstract]) OR "SARS"[Title/Abstract]) OR "sars related coronavirus"[Title/Abstract]) OR "SARS-CoV"[Title/Abstract]) OR "sars associated coronavirus"[Title/Abstract]) OR "SARS-CoV"[Title/Abstract])) OR (((((((((("COVID-19"[Supplementary Concept] OR "severe acute respiratory syndrome coronavirus 2"[Supplementary Concept]) OR "COVID-19"[Title/Abstract]) OR "SARS-CoV-2"[Title/Abstract]) OR "2019 novel coronavirus"[Title/Abstract]) OR "2019-nCoV"[Title/Abstract]) OR "2019-nCoV"[Title/Abstract]) OR "COVID19"[Title/Abstract]) OR "coronavirus disease 2019"[Title/Abstract]) OR "SARS-CoV-2"[Title/Abstract]) OR "wuhan coronavirus"[Title/Abstract]))) AND (((("prevention and control"[MeSH Subheading] AND "prevention and control"[MeSH Subheading]) OR "primary prevention"[MeSH Terms]) OR "prevent*"[Title/Abstract]) OR ((((((((((((((((("therapeutics"[MeSH Terms] OR "drug therapy"[MeSH Terms]) OR "hospitalization"[MeSH Terms]) OR "therapeutic*"[Title/Abstract]) OR "therapy"[Title/Abstract]) OR "treat*"[Title/Abstract]) OR "hospitalization"[Title/Abstract]) OR "icu admission"[Title/Abstract]) OR "intensive care unit admission"[Title/Abstract]) OR "length-of-stay"[Title/Abstract]) OR "length-of-stay"[Title/Abstract]) OR "respiratory distress syndrome, adult"[MeSH Terms]) OR "acute respiratory distress syndrome"[Title/Abstract]) OR "respiratory related complication*"[Title/Abstract]) OR "respiratory related complication*"[Title/Abstract]) OR "mortality"[Title/Abstract]) OR "death*"[Title/Abstract]) OR "mortality"[MeSH Terms]) OR "patient acuity"[MeSH Terms]) OR "sever*"[Title/Abstract]))) NOT ("child*"[Title/Abstract] OR "pediatr*"[Title/Abstract])</td></tr>
<tr><td>Cochrane</td><td><table><tr><th>ID</th><th>Search</th></tr></table></td></tr>
</table>

#1	(deficien*):ti,ab,kw
#2	(supplement*):ti,ab,kw
#3	MeSH descriptor: [Dietary Supplements] this term only
#4	MeSH descriptor: [Micronutrients] explode all trees
#5	(Vitamin):ti,ab,kw OR (mineral*):ti,ab,kw OR (zinc):ti,ab,kw OR (selen*):ti,ab,kw OR (magnesium):ti,ab,kw OR (copper):ti,ab,kw OR (cupr*):ti,ab,kw OR (iron):ti,ab,kw OR (ferric):ti,ab,kw OR (ferrous):ti,ab,kw OR (ferrochel):ti,ab,kw
#6	MeSH descriptor: [Zinc] explode all trees
#7	MeSH descriptor: [Zinc Compounds] explode all trees
#8	MeSH descriptor: [Zinc Acetate] explode all trees
#9	MeSH descriptor: [Orotic Acid] explode all trees
#10	MeSH descriptor: [Selenium] explode all trees
#11	MeSH descriptor: [Selenium Compounds] explode all trees
#12	MeSH descriptor: [Selenocysteine] explode all trees
#13	MeSH descriptor: [Selenomethionine] explode all trees
#14	MeSH descriptor: [Vitamin D] explode all trees
#15	MeSH descriptor: [Carotenoids] explode all trees
#16	MeSH descriptor: [Vitamin E] explode all trees
#17	MeSH descriptor: [Vitamin K] explode all trees
#18	MeSH descriptor: [Ascorbic Acid] explode all trees

	#19	MeSH descriptor: [Thiamine] explode all trees
	#20	MeSH descriptor: [Riboflavin] explode all trees
	#21	MeSH descriptor: [Niacinamide] explode all trees
	#22	MeSH descriptor: [Pantothenic Acid] explode all trees
	#23	MeSH descriptor: [Vitamin B 6] explode all trees
	#24	MeSH descriptor: [Biotin] explode all trees
	#25	MeSH descriptor: [Folic Acid] explode all trees
	#26	MeSH descriptor: [Vitamin B 12] explode all trees
	#27	MeSH descriptor: [Magnesium] explode all trees
	#28	MeSH descriptor: [Magnesium Compounds] explode all trees
	#29	MeSH descriptor: [Copper] explode all trees
	#30	MeSH descriptor: [Copper Sulfate] explode all trees
	#31	MeSH descriptor: [Iron] explode all trees
	#32	MeSH descriptor: [Iron Compounds] explode all trees
	#33	{OR #3-#32}
	#34	(#1 OR #2) AND #33
	#35	MeSH descriptor: [Avitaminosis] explode all trees
	#36	MeSH descriptor: [Magnesium Deficiency] explode all trees
	#37	{OR #34-#36}

	#38	MeSH descriptor: [Quaternary Prevention] explode all trees
	#39	MeSH descriptor: [Tertiary Prevention] explode all trees
	#40	MeSH descriptor: [Secondary Prevention] explode all trees
	#41	MeSH descriptor: [Primary Prevention] explode all trees
	#42	prevent*:ti,ab,kw
	#43	MeSH descriptor: [Therapeutics] explode all trees
	#44	treat*:ti,ab,kw OR severity:ti,ab,kw OR mortality:ti,ab,kw OR death?:ti,ab,kw OR 'length of stay':ti,ab,kw
	#45	respiratory near/2 complication*(:ti,ab,kw)
	#46	MeSH descriptor: [Length of Stay] explode all trees
	#47	MeSH descriptor: [Respiratory System] explode all trees
	#48	complicat*:ti,ab,kw
	#49	#47 AND #48
	#50	MeSH descriptor: [Mortality] explode all trees
	#51	{OR #38-#46, #49-#50}
	#52	MeSH descriptor: [Middle East Respiratory Syndrome Coronavirus] explode all trees
	#53	MeSH descriptor: [SARS Virus] explode all trees
	#54	MeSH descriptor: [Severe Acute Respiratory Syndrome] explode all trees
	#55	MeSH descriptor: [Coronavirus Infections] this term only
	#56	'Middle east respiratory syndrome':ti,ab,kw OR MERS:ti,ab,kw OR MERS-CoV:ti,ab,kw

	#57 'Severe acute respiratory syndrome':ti,ab,kw OR SARS:ti,ab,kw OR SARS-CoV:ti,ab,kw OR 'SARS Associated coronavirus':ti,ab,kw<br>#58 COVID-19:ti,ab,kw OR SARS-CoV-2:ti,ab,kw OR '2019 nCoV':ti,ab,kw OR COVID19:ti,ab,kw OR 'coronavirus disease 2019':ti,ab,kw OR 'Wuhan coronavirus':ti,ab,kw OR 'novel coronavirus 2019':ti,ab,kw<br>#59 ({OR #52-#58}) AND #51<br>#60 #59 AND #37<br>#61 MeSH descriptor: [Child] explode all trees<br>#62 #60 NOT #61
Scopus	((((TITLE-ABS-KEY (deficien*) OR TITLE-ABS-KEY (supplement*)) W/2 (TITLE-ABS-KEY (micronutrient) OR TITLE-ABS-KEY (mineral) OR TITLE-ABS-KEY ("vitamin *") OR TITLE-ABS-KEY (ascorb*) OR TITLE-ABS-KEY ("cevitamic acid") OR TITLE-ABS-KEY (ergocalciferol) OR TITLE-ABS-KEY (cholecalciferol) OR TITLE-ABS-KEY (dihydrotachysterol) OR TITLE-ABS-KEY ("beta carotene") OR TITLE-ABS-KEY (tocopherol) OR TITLE-ABS-KEY (tocotrienol) OR TITLE-ABS-KEY (thiamine) OR TITLE-ABS-KEY (riboflavin) OR TITLE-ABS-KEY (niacinamide) OR TITLE-ABS-KEY ("Pantothenic acid") OR TITLE-ABS-KEY (biotin) OR TITLE-ABS-KEY ("folic acid") OR TITLE-ABS-KEY (zinc) OR TITLE-ABS-KEY (selen*) OR TITLE-ABS-KEY (magnesium) OR TITLE-ABS-KEY (copper) OR TITLE-ABS-KEY (iron))) AND (TITLE-ABS-KEY (covid-19) OR TITLE-ABS-KEY ("severe acute respiratory syndrome coronavirus 2") OR TITLE-ABS-KEY (sars-cov-2) OR TITLE-ABS-KEY ("2019 novel coronavirus") OR TITLE-ABS-KEY ("2019-novel coronavirus") OR TITLE-ABS-KEY (2019-ncov) OR TITLE-ABS-KEY ("coronavirus disease 2019") OR TITLE-ABS-KEY ("Wuhan coronavirus") OR TITLE-ABS-KEY ("Severe Acute Respiratory Syndrome") OR TITLE-ABS-KEY ("SARS Virus") OR TITLE-ABS-KEY ("Severe Acute Respiratory Syndrome Virus") OR TITLE-ABS-KEY ("SARS") OR TITLE-ABS-KEY ("SARS-Related Coronavirus") OR TITLE-ABS-KEY (sars-cov) OR TITLE-ABS-KEY ("SARS-Associated Coronavirus") OR TITLE-ABS-KEY ("Middle East Respiratory Syndrome Coronavirus") OR TITLE-ABS-KEY (mers) OR TITLE-ABS-KEY (mers-cov) OR TITLE-ABS-KEY ("Middle East respiratory syndrome related coronavirus") OR TITLE-ABS-KEY ("Middle East respiratory syndrome-related coronavirus") OR TITLE-ABS-KEY ("Middle East respiratory syndrome"))) AND (TITLE-ABS-KEY (prevent*) OR TITLE-ABS-KEY (therap*) OR TITLE-ABS-KEY (hospitalization) OR TITLE-ABS-KEY (treat*) OR (TITLE-ABS-KEY (icu) W/3 TITLE-ABS-KEY (admi*)) OR (TITLE-ABS-KEY ("intensive care unit") W/3 TITLE-ABS-KEY (admission)) OR TITLE-ABS-KEY (length-of-stay) OR TITLE-ABS-KEY ("length of stay") OR (TITLE-ABS-KEY (progress*) W/2 TITLE-ABS-KEY ("respiratory complication*")) OR TITLE-ABS-KEY (mortality) OR TITLE-ABS-KEY (death?) OR TITLE-ABS-KEY ("Patient Acuity") OR TITLE-ABS-KEY (sever*))) AND NOT (TITLE-ABS-KEY (animal?) OR TITLE-ABS-KEY (cell?) OR TITLE-ABS-KEY (plant?) OR TITLE-ABS-KEY (agri*) OR TITLE-ABS-KEY (child*))

CINAHL	S1	(MH "Ascorbic Acid Deficiency") OR (MM "Avitaminosis") OR (MH "Vitamin A Deficiency") OR (MH "Folic Acid Deficiency") OR (MH "Riboflavin Deficiency") OR (MH "Thiamine Deficiency") OR (MH "Vitamin B6 Deficiency") OR (MH "Vitamin B12 Deficiency") OR (MH "Vitamin D Deficiency") OR (MH "Vitamin E Deficiency") OR (MH "Vitamin K Deficiency") OR (MH "Iodine Deficiency")
	S2	(MH "Iron Compounds+") OR (MH "Selenium") OR (MH "Selenium Compounds") OR (MH "Zinc Compounds+") OR (MH "Vitamins+")
	S3	TI supplement* OR TI deficien* OR AB supplement* OR AB deficien*
	S4	TI vitamin * OR TI mineral OR AB vitamin * OR AB mineral OR TI zinc OR TI selenium OR TI magnesium OR TI copper OR TI iron OR TI micronutrient OR AB micronutrient
	S5	AB zinc OR AB selenium OR AB magnesium OR AB copper OR AB iron
	S6	AB zinc supplementation OR AB selenium supplementation OR AB magnesium supplementation OR AB iron supplementation
	S7	TI zinc supplementation OR TI selenium supplementation OR TI magnesium supplementation OR TI iron supplementation
	S8	(S4 or S5 or S2) AND S3
	S9	S1 or S6 or S7 or S8
	S10	TI (covid-19 or "2019-novel coronavirus" or 2019-ncov or SARS-CoV-2 or COVID19 or "2019 nCoV" or "novel coronavirus 2019" or "2019 novel coronavirus" or "wuhan coronavirus" or "SARS CoV-2") OR AB (covid-19 or "2019-novel coronavirus" or 2019-ncov or SARS-CoV-2 or COVID19 or "2019 nCoV" or "novel coronavirus 2019" or "2019 novel coronavirus" or "wuhan coronavirus" or "SARS CoV-2")
	S11	TI (SARS or "severe acute respiratory syndrome" or "severe acute respiratory syndrome coronavirus" or SARS-CoV or "SARS virus" or "SARS-related coronavirus" or "SARS-associated coronavirus" or "SARS related coronavirus" or "SARS associated coronavirus") OR AB (SARS or "severe acute respiratory syndrome" or "severe acute respiratory syndrome coronavirus" or SARS-CoV or "SARS virus" or "SARS-related coronavirus" or "SARS-associated coronavirus" or "SARS related coronavirus" or "SARS associated coronavirus")
	S12	TI (MERS or "middle east respiratory syndrome" or "middle east respiratory syndrome coronavirus" or MERS-CoV or "MERS virus" or "MERS-related coronavirus" or "MERS-associated coronavirus" or "middle east respiratory syndrome related coronavirus" or "middle east respiratory syndrome associated coronavirus") OR AB (MERS or "middle east respiratory syndrome" or "middle east respiratory syndrome coronavirus" or MERS-CoV or "MERS virus" or "MERS-related coronavirus" or "MERS-associated coronavirus" or "middle east respiratory syndrome related coronavirus" or "middle east respiratory syndrome associated coronavirus")
	S13	(MH "Coronavirus Infections+/DT/PC/RF")

	S14 | (S10 or S11 or S12 or S13) AND S9
Embase	(('severe acute respiratory syndrome'/exp OR 'sars':ti,ab OR 'sars-related coronavirus'/exp OR 'sars-like cov':ti,ab OR 'sars-like coronavirus':ti,ab OR 'sars-related cov':ti,ab OR 'sars-related coronavirus':ti,ab OR 'sarsr-cov':ti,ab OR 'severe acute respiratory syndrome-like coronavirus':ti,ab OR 'severe acute respiratory syndrome-related coronavirus':ti,ab OR sars:ti,ab OR 'sars cov':ti,ab OR 'severe acute respiratory syndrome':ti,ab OR 'middle east respiratory syndrome coronavirus'/exp OR 'mers coronavirus' OR 'mers virus' OR 'mers-cov' OR 'middle east respiratory syndrome coronavirus' OR 'middle east respiratory syndrome'/exp OR 'mers coronavirus infection' OR 'mers infection' OR 'mers virus infection' OR 'middle east respiratory syndrome' OR 'middle east respiratory syndrome coronavirus infection' OR 'mers cov':ti,ab OR 'middle east respiratory syndrome':ti,ab OR 'coronavirus disease 2019'/exp OR '2019-ncov disease':ti,ab OR '2019-ncov infection':ti,ab OR 'covid 19':ti,ab OR 'covid 2019':ti,ab OR 'wuhan coronavirus disease':ti,ab OR 'wuhan coronavirus infection':ti,ab OR 'coronavirus disease 2019':ti,ab OR 'ncov 2019 disease':ti,ab OR 'ncov 2019 infection':ti,ab OR 'novel coronavirus 2019 disease':ti,ab OR 'novel coronavirus 2019 infection':ti,ab OR 'novel coronavirus disease 2019':ti,ab OR 'novel coronavirus infection 2019':ti,ab OR 'sars cov 2':ti,ab OR 'sars-cov 2':ti,ab) AND (('micro-element deficiency' OR 'micro-elements deficiency' OR 'microelement deficiency' OR 'mineral deficiency' OR 'minerals deficiency' OR 'trace element deficiency' OR 'trace element deficit' OR 'trace elements deficiency' OR 'vitamin deficiency'/exp OR 'avitaminosis' OR 'corrinoid deficiency' OR 'deficiency, vitamin' OR 'hypo-vitaminosis' OR 'hypovitaminosis' OR 'multivitamin deficiency' OR 'vit. deficiency' OR 'vitamin deficiency' OR 'vitamins deficiency' OR 'mineral supplementation'/exp OR 'mineral supplementation' OR 'vitamin supplementation'/exp OR 'supplementation, vitamin' OR 'vitamin supplementation' OR 'vitamin d supplementation'/exp OR 'iron supplementation'/exp) OR (('supplementation'/exp OR supplement*:ti,ab OR deficien*:ti,ab) AND (mineral*:ti,ab OR 'zinc'/exp OR zinc:ti,ab OR 'selenium'/exp OR selenium:ti,ab OR 'selenite'/exp OR 'selenate'/exp OR 'selenomethionine'/exp OR 'selenocysteine'/exp OR 'selenium yeast'/exp OR 'magnesium'/exp OR magnesium:ti,ab OR 'magnesium oxide'/exp OR 'magnesium carbonate'/exp OR 'copper'/exp OR copper:ti,ab OR 'iron'/exp OR iron:ti,ab OR 'iron glycinate'/exp OR 'iron derivative'/exp OR ferrous:ti,ab OR ferric:ti,ab OR vitamin*:ti,ab OR 'vitamin d'/exp OR 'retinol'/exp OR 'beta carotene'/exp OR 'alpha tocopherol'/exp OR 'alpha tocotrienol'/exp OR 'vitamin k group'/exp OR 'vitamin b group'/exp OR 'ascorbic acid'/exp))) AND ((('intensive care unit'/exp OR 'gicu' OR 'gicus' OR 'icu`s' OR 'close attention unit' OR 'combined medical and surgical icu' OR 'combined surgical and medical icu' OR 'critical care unit' OR 'general icu' OR 'intensive care department' OR 'intensive care unit' OR 'intensive care units' OR 'intensive therapy unit' OR 'intensive treatment unit' OR 'medical-surgery icu' OR 'medical/surgical icu' OR 'medical/surgical icus' OR 'medico-surgical icu' OR 'mixed medical and surgical icu' OR 'mixed surgical and medical icu' OR 'respiratory care unit' OR 'respiratory care units' OR 'special care unit' OR 'surgery/medical icu' OR 'surgical-medical icus' OR 'surgical/medical icu' OR 'unit, intensive care') AND ('hospital admission'/exp OR 'admission, hospital' OR 'admitting department, hospital' OR 'hospital admission' OR 'hospital admittance' OR 'patient admission') OR ('intensive care unit' NEAR/2 admission)) OR severity:ti,ab OR 'hospital admission'/exp OR 'disease severity'/exp OR 'length of stay'/exp OR 'prevention and control'/exp OR prevent*:ti,ab OR 'therapy effect'/exp OR treat*:ti,ab OR therapy:ti,ab OR ('complication'/exp AND respira*:ti,ab))) NOT (child*:ti,ab OR pediatric*:ti,ab)
