# Supplementary figures and images for: Micronutrients Deficiency, Supplementation and Novel Coronavirus Infections—A Systematic Review and Meta-Analysis
Source: Nutrients. 2021 May 10;13(5):1589. doi: 10.3390/nu13051589 (PMC8151981; doi:10.3390/nu13051589)

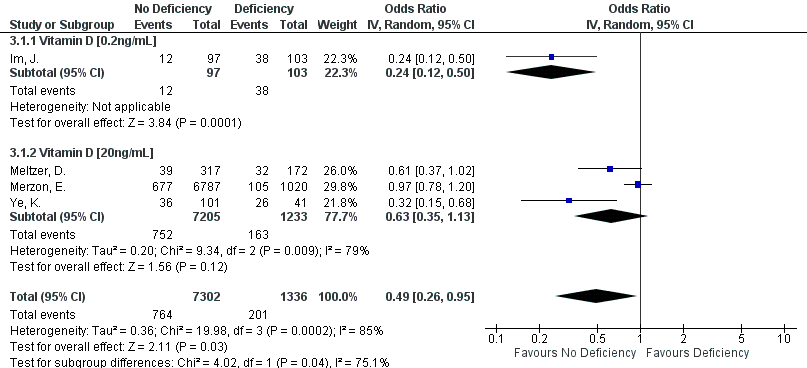

Supplement: Supplementary file 1 [file nutrients-13-01589-s001.zip › Supplementary Tables and Figures/Figure S1.png]

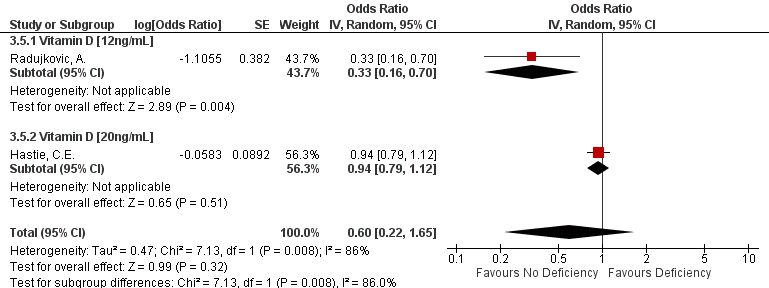

Supplement: Supplementary file 1 [file nutrients-13-01589-s001.zip › Supplementary Tables and Figures/Figure S10.png]

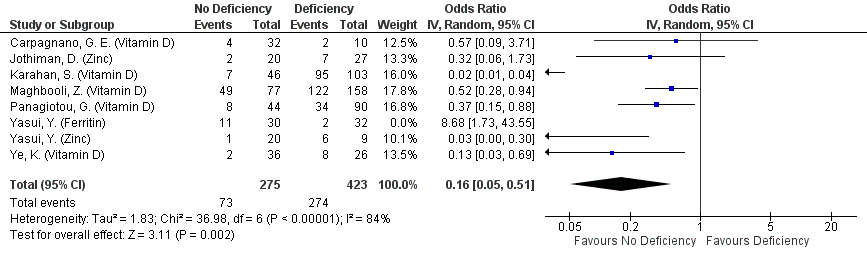

Supplement: Supplementary file 1 [file nutrients-13-01589-s001.zip › Supplementary Tables and Figures/Figure S11.png]

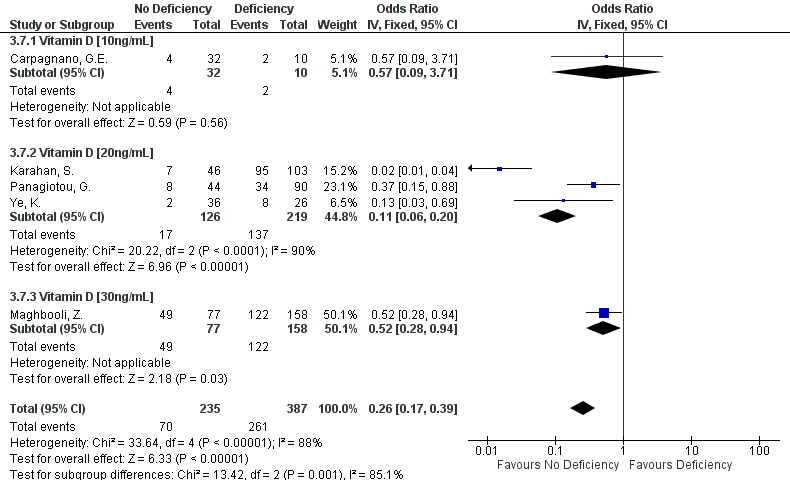

Supplement: Supplementary file 1 [file nutrients-13-01589-s001.zip › Supplementary Tables and Figures/Figure S12.png]

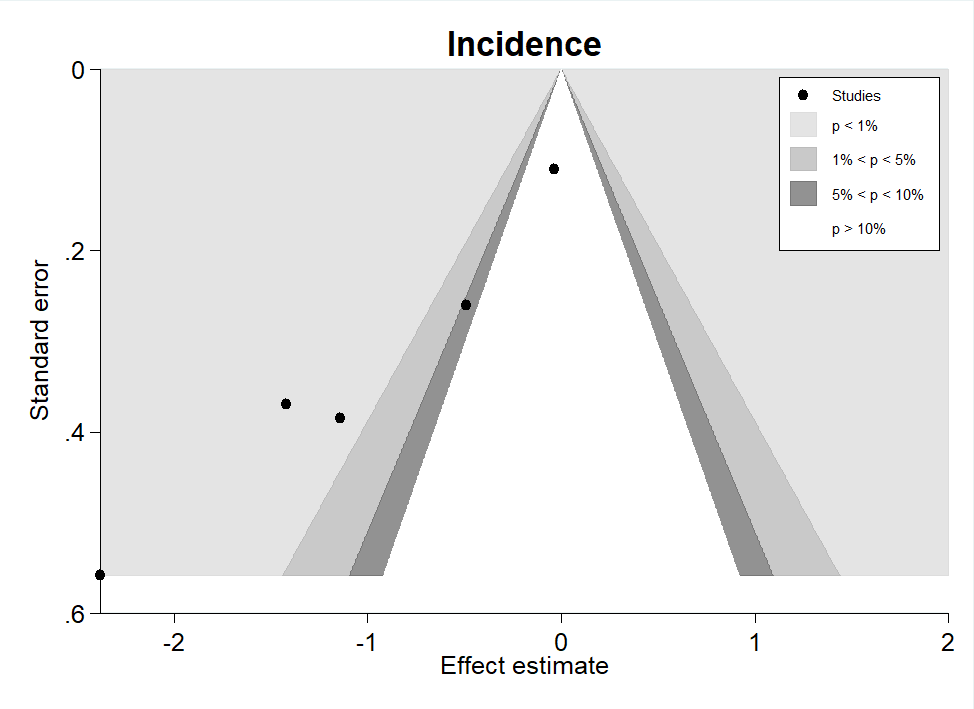

Supplement: Supplementary file 1 [file nutrients-13-01589-s001.zip › Supplementary Tables and Figures/Figure S13A.png]

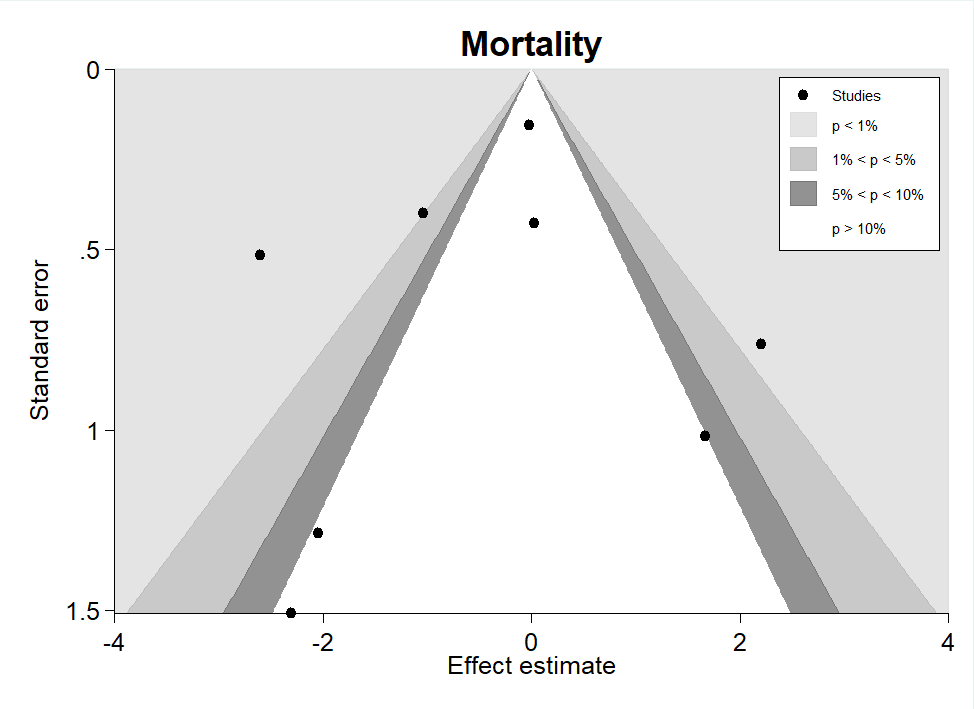

Supplement: Supplementary file 1 [file nutrients-13-01589-s001.zip › Supplementary Tables and Figures/Figure S13B.png]

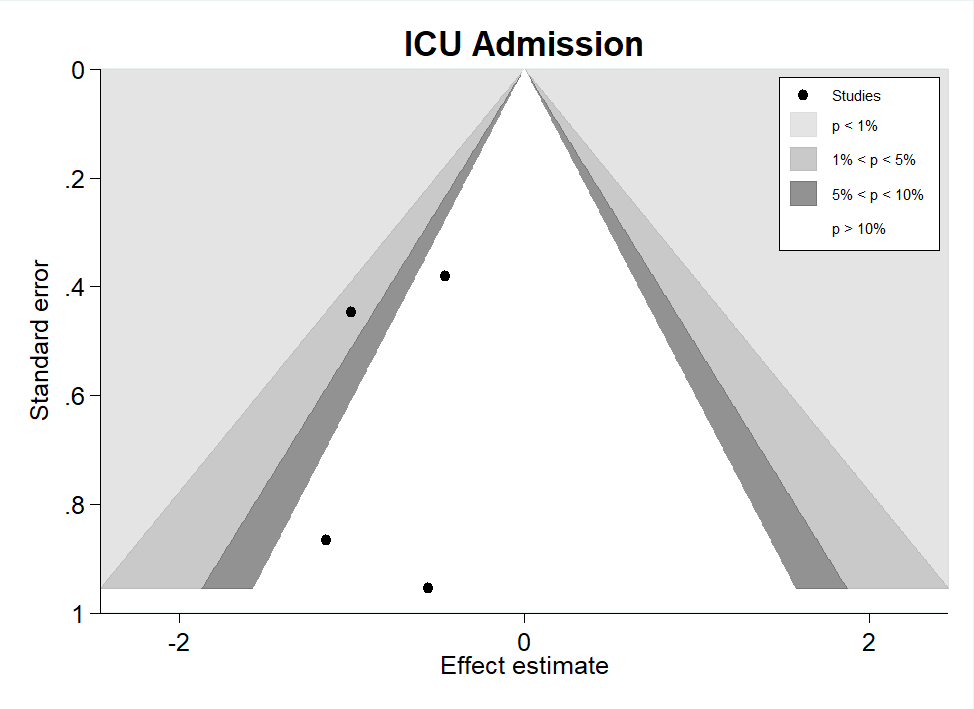

Supplement: Supplementary file 1 [file nutrients-13-01589-s001.zip › Supplementary Tables and Figures/Figure S13C.png]

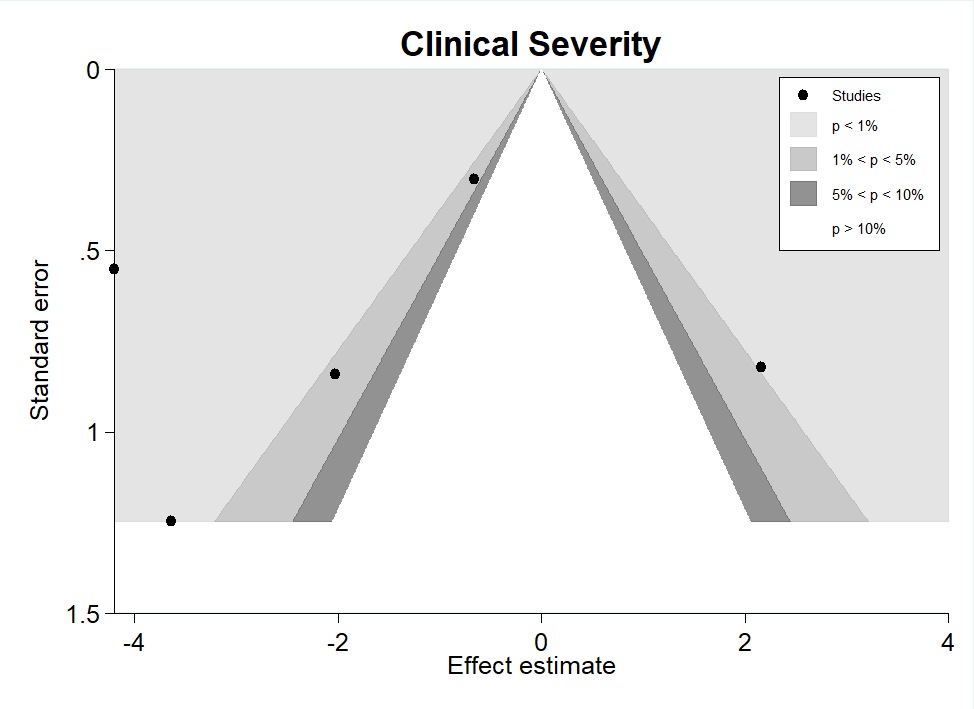

Supplement: Supplementary file 1 [file nutrients-13-01589-s001.zip › Supplementary Tables and Figures/Figure S13D.png]

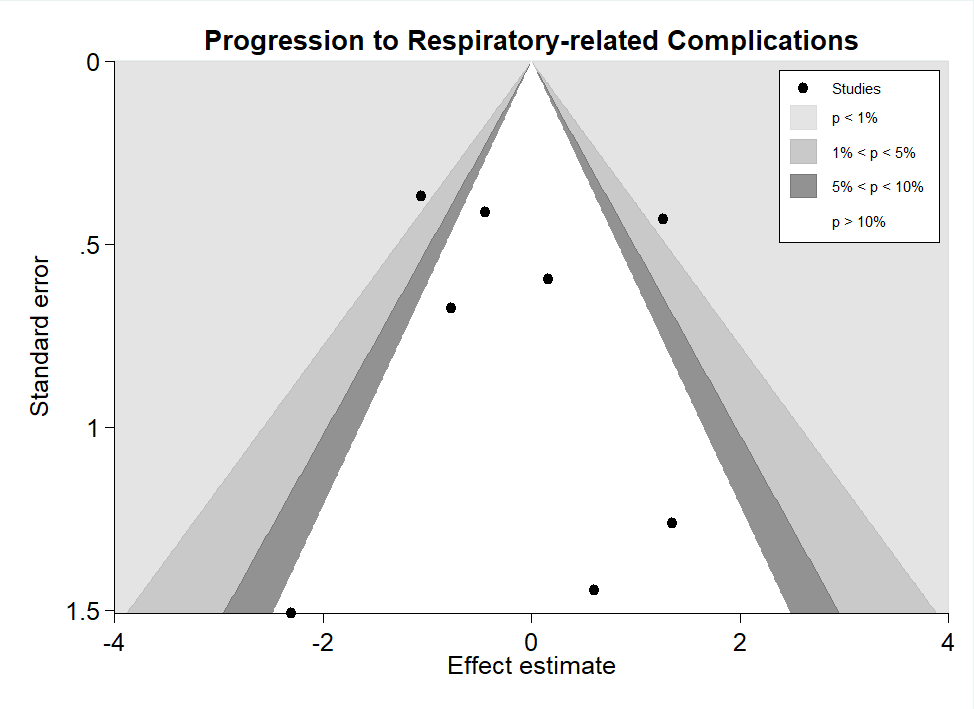

Supplement: Supplementary file 1 [file nutrients-13-01589-s001.zip › Supplementary Tables and Figures/Figure S13E.png]

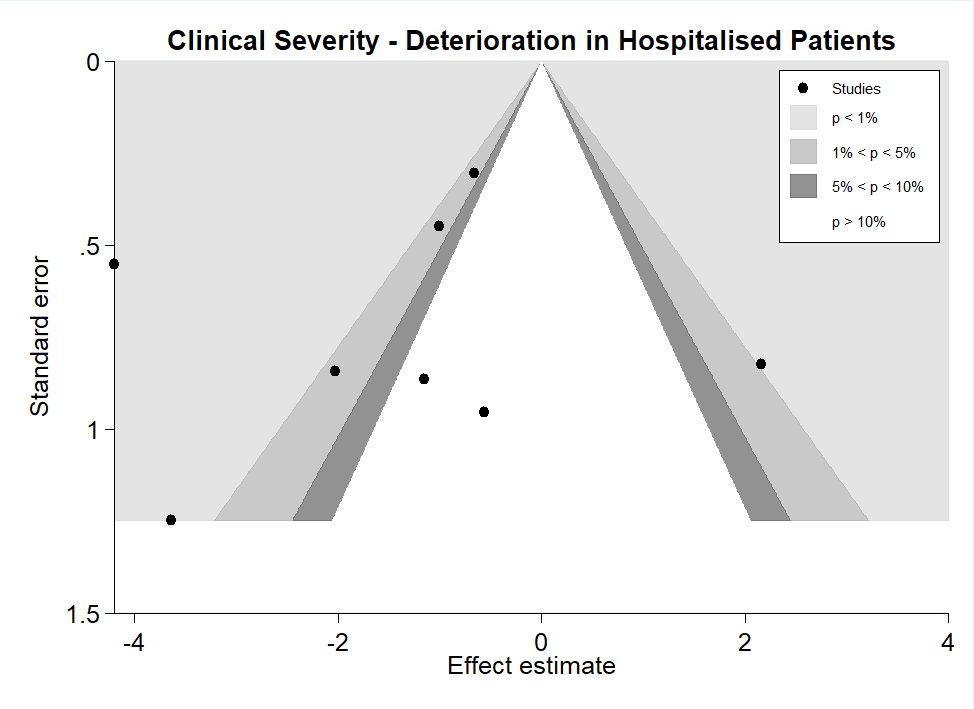

Supplement: Supplementary file 1 [file nutrients-13-01589-s001.zip › Supplementary Tables and Figures/Figure S13F.png]

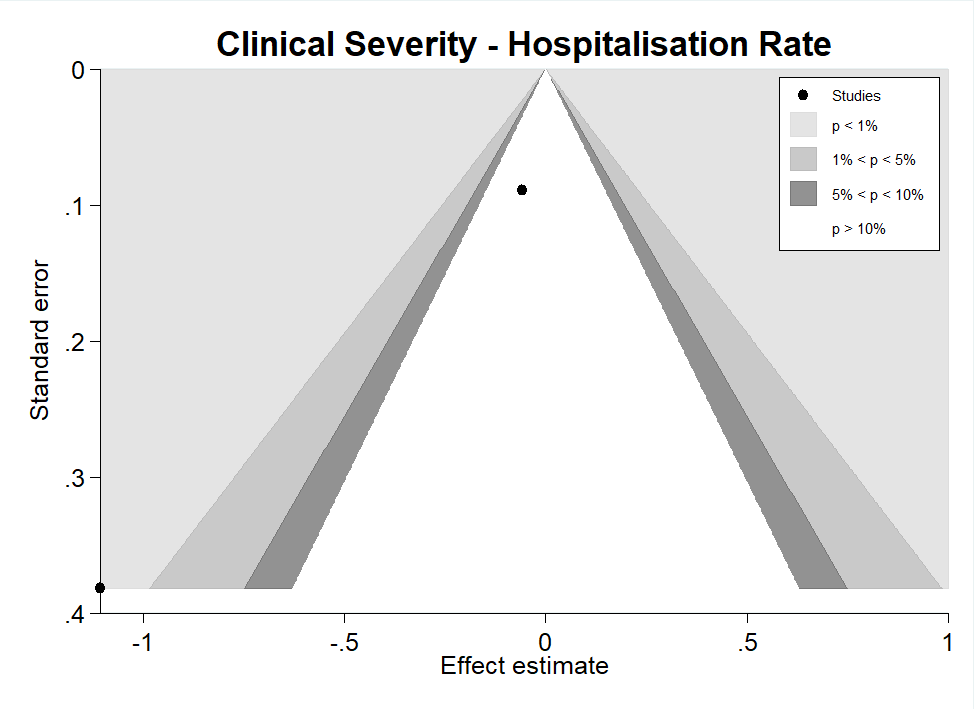

Supplement: Supplementary file 1 [file nutrients-13-01589-s001.zip › Supplementary Tables and Figures/Figure S13G.png]

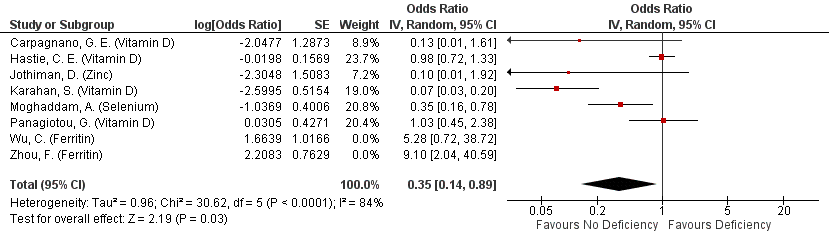

Supplement: Supplementary file 1 [file nutrients-13-01589-s001.zip › Supplementary Tables and Figures/Figure S2.png]

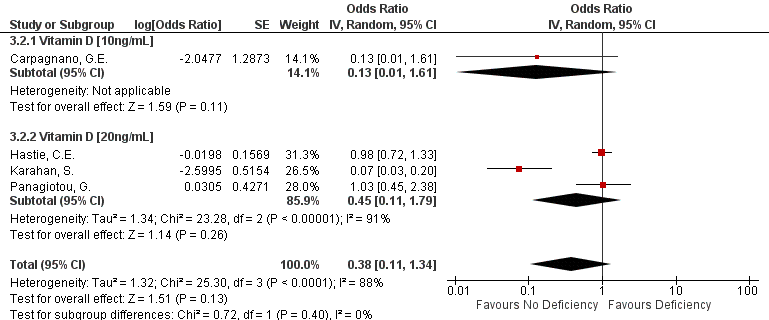

Supplement: Supplementary file 1 [file nutrients-13-01589-s001.zip › Supplementary Tables and Figures/Figure S3.png]

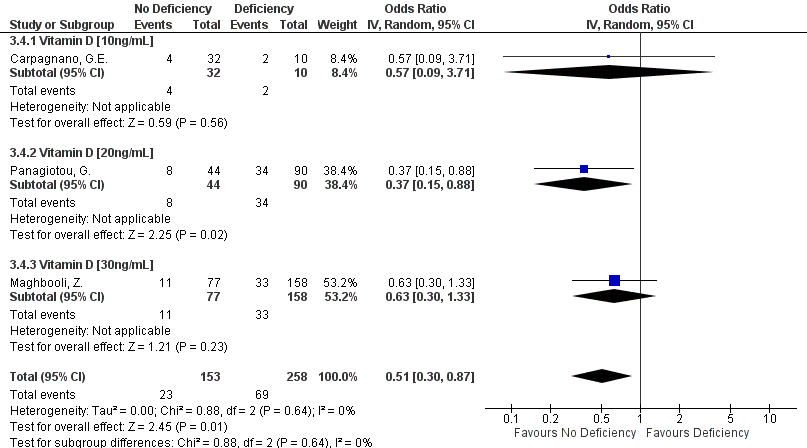

Supplement: Supplementary file 1 [file nutrients-13-01589-s001.zip › Supplementary Tables and Figures/Figure S4.png]

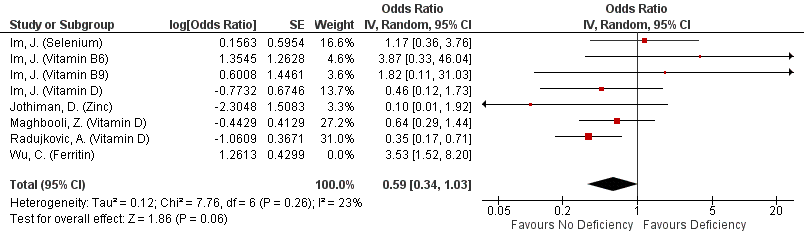

Supplement: Supplementary file 1 [file nutrients-13-01589-s001.zip › Supplementary Tables and Figures/Figure S5.png]

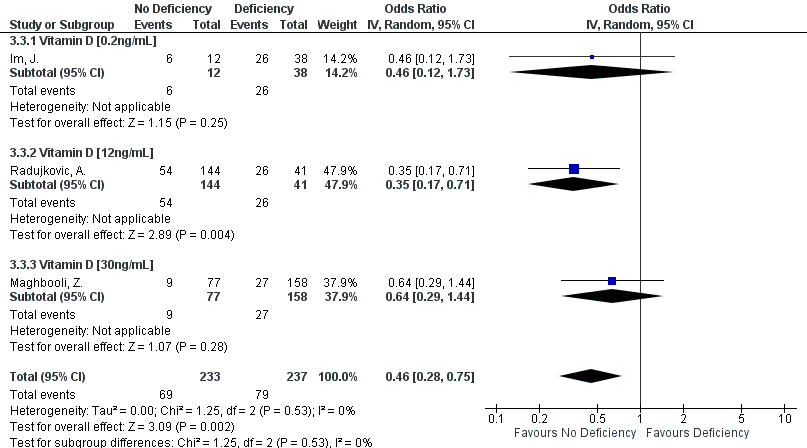

Supplement: Supplementary file 1 [file nutrients-13-01589-s001.zip › Supplementary Tables and Figures/Figure S6.png]

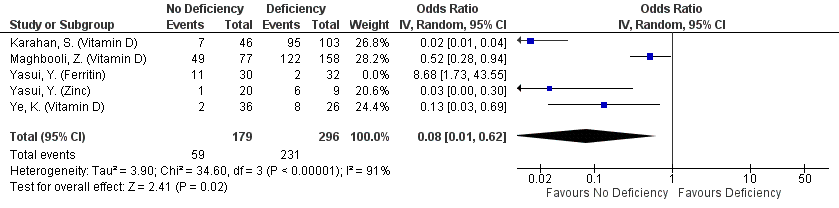

Supplement: Supplementary file 1 [file nutrients-13-01589-s001.zip › Supplementary Tables and Figures/Figure S7.png]

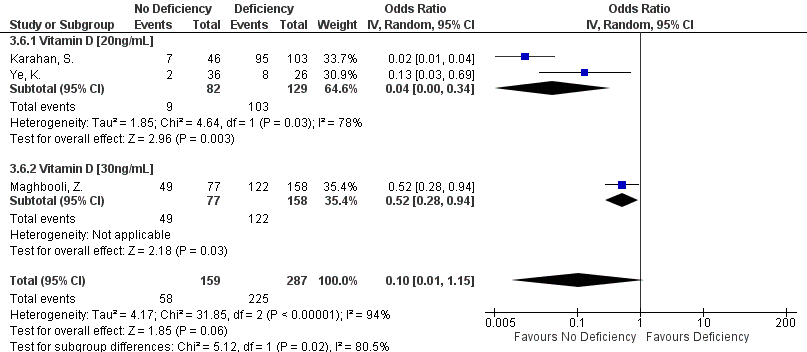

Supplement: Supplementary file 1 [file nutrients-13-01589-s001.zip › Supplementary Tables and Figures/Figure S8.png]

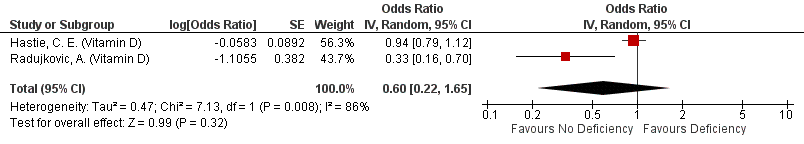

Supplement: Supplementary file 1 [file nutrients-13-01589-s001.zip › Supplementary Tables and Figures/Figure S9.png]
